# A sea of need: provider accounts of strategies used to manage admission demands to safer opioid supply programs in Ontario

**DOI:** 10.1186/s12954-025-01344-3

**Published:** 2025-11-26

**Authors:** Carol Strike, Katherine Rudzinski, Rose A. Schmidt, Gillian Kolla, David Kryszajtys, Melissa Perri, Nat Kaminski, Adrian Guta

**Affiliations:** 1 Dalla Lana School of Public Health, University of Toronto, 155 College St Room 500, Toronto, ON M5T 3M7 Canada; 2 Faculty of Medicine , Memorial University , St. John’s, Canada; 3 School of Social Work, University of Windsor, Windsor, Canada

## Abstract

**Background:**

Since 2016, over 50,928 people have died of an opioid-related overdose in Canada. The unregulated supply of drugs is increasingly toxic and volatile, and fentanyl from unregulated, street-based markets is driving this epidemic. Concerns that existing overdose prevention approaches were insufficient to address the rising number of overdoses led to the implementation of safer supply programs (SSPs) in Canada. SSPs provide prescribed medications to people who use drugs and are designed for individuals at high risk of overdose for whom existing care options have been ineffective or inappropriate. Evidence of SSP impact is growing but implementation processes, including admissions, are not well understood nor well-described in practice guidelines. Our purpose was to describe how the admission processes of four Ontario SSPs evolved and how these changes influenced program reach and perceived effectiveness.

**Methods:**

During 2021, we conducted short demographic and semi-structured interviews with healthcare providers (n = 21) from four SSPs in Ontario about implementation processes, challenges, and impacts. Thematic analysis of data concerning admission processes was conducted in MAXQDA and descriptive statistics in SPSSv28.

**Results:**

Although the desire was for SSPs to have a broad reach, programs quickly realized they needed to develop strategies to manage the high demand for their programs. To manage this demand, strategies were implemented like waitlists, which were later replaced by points-based admission criteria. These admission criteria evolved over time, leading to a client population with high medical and social needs. The combination of high-acuity clients, limited capacity, and funding constraints, exacerbated by COVID-19, caused significant distress and burnout among service providers, prompting further changes to the SSPs.

**Discussion:**

The implementation of SSPs in Ontario highlights the challenges of addressing intersecting public health emergencies in a resource-constrained healthcare system. SSPs, were adaptive and evolved in real time; while these adaptations addressed significant equity gaps, they also underscored the limitations of operating within an under-funded primary care model. The narrowing of admission criteria, necessitated by overwhelming demand and limited resources, ultimately constrained their reach and potential population-level impact.

## Background

Since 2016, over 50,928 people have died of an opioid-related overdose in Canada [1]. The unregulated supply of drugs in Canada is increasingly toxic and volatile, and fentanyl from unregulated, street-based markets is driving this epidemic [1]. In Ontario, fentanyl is responsible for 87% of opioid-related mortality [2]. However, drug market volatility includes fentanyl analogues (e.g., carfentanil), non-medical benzodiazepines (norfludiazepam), and sedatives (e.g., xylazine) [3–7]. Concerns that existing overdose prevention approaches—such as naloxone distribution, supervised injection services, and opioid agonist therapy (OAT)—were insufficient to address the rising number of overdoses led to the implementation of safer supply programs (SSPs) in Canada. The combination of opioids and other adulterants has resulted in atypical overdoses, which are difficult to reverse, even with multiple administrations of naloxone [8]. In response, there have been calls for alternatives to the toxic drug supply. Safer supply is defined by Health Canada as services that “provide prescribed medications to people who use drugs, overseen by a health care practitioner, with the goal of preventing overdoses and saving lives” [9]. SSPs are designed for individuals at high risk of overdose for whom existing care options have been ineffective or inappropriate [9].

In response to the escalating overdose crisis, drug users, activists, and researchers began advocating for a “safe supply” of substances with known dose and composition for people reliant on the volatile unregulated market. The first safe supply program opened in 2016, in London, Ontario [10], but the earlier use of prescribed alternatives to the unregulated supply has been documented in hospitals [11]. In their 2019 concept document, the Canadian Association of People Who Use Drugs called for a range of safe supply models, including prescriber-based programs offering pharmaceutical-grade opioids, stimulants, or benzodiazepines, as well as grassroots, non-medicalized approaches like compassion clubs or buyers’ clubs [12]. Compassion clubs are community-based organizations that provide safe, affordable access to therapeutic substances—especially medical cannabis—through low-barrier, harm-reduction approaches with personalized care and support [13]. Buyers’ clubs are cooperatives that pool resources to access expensive or hard-to-obtain medications, often operating outside formal systems to meet urgent health needs, especially in marginalized communities [14]. The SSPs in this study represent a prescriber-based, medicalized model of safer supply. These programs build on research demonstrating the effectiveness of injectable diacetylmorphine (pharmaceutical heroin) and hydromorphone for people with treatment-refractory opioid use disorder [15–17], as well as the experiential knowledge of clinicians prescribing safer supply before its formal expansion [18].

SSPs differ across Canada but most share common features: 1) daily dispensed, immediate-release opioid tablets (e.g., hydromorphone) mostly for unwitnessed take-home dosing; 2) a longer-acting opioid like slow-release oral morphine (brand name Kadian) or methadone, usually dispensed daily under supervision; and 3) a harm-reduction philosophy guiding service delivery. Some SSPs also prescribe fentanyl (as patches or injectables), stimulants, or benzodiazepines [19]. Compared to other substance use treatments, SSPs offer a more flexible, less clinical approach [20]. For example, take-home doses of short-acting opioids allow participants to manage the timing, method, and route of administration (e.g., injecting or swallowing).

In Ontario, SSPs primarily operate in community health centres with some provided by specialty health care clinics and non-governmental organizations. In addition to prescribing safer supply, these programs deliver comprehensive primary care, including testing and treatment for infectious diseases such as Human Immunodeficiency Virus (HIV) and Hepatitis C (HCV), wound care, and sexual health services. However, SSPs face significant challenges due to operating in underfunded organizations and the overwhelming demand for services among marginalized populations [21, 22].

SSPs are an emergent intervention to an ongoing crisis. A few prescribers in Canada were prescribing safe supply before the Coronavirus Disease 2019 (COVID-19) pandemic, but the pandemic accelerated their development to support physical distancing and stay-at-home measures [18, 22]. Funding from Health Canada’s Substance Use and Addiction Program (SUAP), received in 2020, allowed for the creation of several new pilot SSPs and the expansion of existing ones. By 2023, 28 SSPs were operating with pilot funding from Health Canada [23, 24].

Research on the impact of SSPs is growing [25] and evidence indicates people enrolled in SSPs self-report reduced use of unregulated fentanyl, fewer non-fatal overdoses, and less frequent drug injections [18, 22, 26–35]. Clients and service providers also report improvements in clients’ physical health, mental health, housing stability, income, and access to primary care [28–33, 36]. Using administrative data, Gomes, Kolla [18] demonstrated that clients of an SSP in London, Ontario had reduced emergency department visits, hospital admissions, infectious complications and lower healthcare costs compared to a matched control group not receiving safer supply. Similarly, a study from British Columbia showed a significant reduction in opioid-related overdose mortality among 5,882 people receiving safer supply compared to matched controls [37].

Despite promising outcomes, there are fewer reports describing the implementation of SSPs. SSPs are an example of a “complex intervention” [38]. They are characterized by multiple components (e.g., outreach, intake processes, treatments), target behaviours (e.g., drug use patterns, overdose prevention, infectious disease reduction), and the need for varied expertise (e.g., physicians, nurses, health navigators) [38, 39]. Complex interventions are rarely fixed or static; instead, they adapt to local contexts through the application of multiple forms of evidence and knowledge [40]. In Ontario, SSPs were implemented during the rapidly evolving crises of the COVID-19 pandemic and escalating opioid-related overdoses, and understanding how interventions like SSPs interact with and are shaped by their context is essential to understanding program adaptation, implementation, and scale-up over time [39].

Since 2018, Canadian guidelines for implementing and operating SSPs [41, 42] and professional guidance on prescribing opioids like slow-release oral morphine as an alternative for treating opioid use disorder [43–45] have expanded. However, these guidelines offer limited direction on admission processes, focusing instead on eligibility criteria such as high overdose risk and prior enrolment in OAT. In this paper, our purpose was to describe how the admission processes of four Ontario SSPs evolved and how these changes influenced program reach and perceived effectiveness.

## Methods

This paper draws on interviews from a qualitative study evaluating the implementation and outcomes of four SSPs in Ontario: three in Toronto and one in London. Details on SSPs and methods can be found in Schmidt, Kaminski [33] and Gagnon, Rudzinski [34]. Our study was designed to collect data on the following domains from the Consolidated Framework for Implementation Research (CFIR): innovation, the outer setting (economic and political climate), the inner setting (intra-organizational structure), characteristics of individuals involved, and the process of program implementation [36]. These CFIR constructs were used to develop the semi-structured interview guide.

Demographic and professional data were collected via an interviewer-administered online questionnaire (Table 1). Service providers were recruited through organizational email lists, which included all SSP prescribers and through snowball sampling. Between February 26 and July 11, 2021, we conducted 21 interviews by phone or Zoom, lasting 40–105 min (median 59 min). Participants provided verbal consent; interviews were recorded, transcribed, and checked for accuracy. Nurses and allied health professionals received a $40 honorarium (by e-transfer); prescribers were not provided an honorarium. Ethics approval was obtained from the University of Toronto Research Ethics Board (Protocol: 40140).

**Table 1:** Service provider demographics and practice history

		N	%
*Profession*			
	Nurse practitioner	5	23.8%
	Registered nurse	5	23.8%
	Physician	4	19.0%
	Community Health Worker	2	9.5%
	Client care support, SSP program administrator	1	4.8%
	Health/systems navigator	2	9.5%
	Outreach worker/site coordinator	1	4.8%
	SSP Case manager	1	4.8%
*Site*			
	Parkdale queen west community health centre	9	42.9%
	Intercommunity health centre	6	28.6%
	South riverdale community health centre	3	14.3%
	Street health	3	14.3%
*Gender*			
	Woman	13	61.9%
	Man	6	28.6%
	Nonbinary	2	9.5%
*Race/ethnicity*			
	White	15	71.4%
	East Asian	2	9.5%
	Indigenous hispanic	1	4.8%
	Mixed	1	4.8%
	Southeast Asian	1	4.8%
	Black	1	4.8%
Age (category)*			
	25–29	1	4.8%
	30–34	7	33.3%
	35–39	5	23.8%
	40–44	3	14.3%
	45–49	3	14.3%
	55–59	1	4.8%
*Years worked- at site*			
	Less than 6 months	1	4.8%
	6–11 months	5	23.8%
	1–2 years	3	14.3%
	3–5 years	5	23.8%
	6–10 years	6	28.6%
	More than 10 years	1	4.8%
*Years worked- HIV*			
	1–2 years	1	4.8%
	3–5 years	4	19.0%
	5–10 years	1	4.8%
	6–10 years	9	42.9%
	More than 10 years	6	28.6%
*Years worked- PWUD*			
	1–2 years	1	4.8%
	3–5 years	3	14.3%
	5–10 years	1	4.8%
	6–10 years	10	47.6%
	More than 10 years	6	28.6%
*Hours worked*			
	20–34	2	9.5%
	35–39	10	47.6%
	40–44	5	23.8%
	45–49	2	9.5%
	More than 55	2	9.5%
*Number of continuing education courses on substance use or harm reduction *			
	None	3	14.3%
	1	3	14.3%
	2	6	28.6%
	3	7	33.3%
	5	2	9.5%

*Note one practitioner did not provide their age

The present analysis focuses on the implementation process (e.g., reach, adaptation), mechanisms of impact, context, and outcomes [46] from the perspective of SSP prescribers (physicians, nurse practitioners), nurses, and allied health workers (e.g., community health workers, administrators). Descriptive analysis of demographic data was conducted using SPSS version 28 (IBM) and qualitative analysis was managed with MAXQDA. A collaboratively developed codebook, based on key concepts from the CFIR [47, 48], was refined with emergent codes added until the team agreed on a stable set of codes. Using a thematic analytical approach [49, 50] each interview was coded by one team member and checked by another for accuracy. Disagreements were resolved through consensus and additional codes were added if collectively deemed necessary. For this manuscript, analytic memos were written based on coded data related to admission procedures and implementation issues: policies, guidelines and regulations; reach; priority groups; “manipulation” of admission criteria by providers; program size; waitlist and discharges. Below, we include direct quotes from the interviews to substantiate and animate the analysis. Each quote includes a code to the original transcript (study site- interview number, type of participant). For example, SSP 1-102, Prescriber-NP refers to safe supply program 1, interview #102, participant = safe supply prescriber who is a nurse practitioner).

## Results

Results of our analysis indicated that although they desired to have a broad reach, programs quickly realized they needed to develop strategies to manage the high demand for their programs. To manage this demand, the programs implemented strategies like waitlists, which were later replaced by points-based admission criteria. These admission criteria evolved over time, leading to a client population with high medical and social needs. The combination of high-acuity clients, limited capacity, and funding constraints, exacerbated by COVID-19, caused significant distress and burnout among service providers, prompting further changes to the SSPs.

### Managing high demand: referrals and waitlists

In the interviews, service providers explained that the programs were initially designed to address high rates of fentanyl-related overdoses among people with a history of opioid overdose, a history of previous unsuccessful attempts of traditional OAT (i.e., methadone, buprenorphine treatment) and imminent risk of overdose. At inception, the programs had targeted admissions because, as one allied health worker remarked, “*We’d love to take anyone but when we do have to prioritize, we look at who’s at immediate risk of death*” (SSP 1-202, Program admin). However, in the face of immense demand, prescribers described having to develop more specific admission guidelines, as the criteria ‘imminent risk of overdose-related death’ could “*theoretically include everyone who uses street-level drugs, but we had to find a way to prioritize, just because the need is so big*” (SSP 1-102, Prescriber, NP).

At the time of the study, all programs had received funding from the Federal Government to operate as pilot programs and they quickly discovered that the funding was not sufficient to meet the demand in the community. One interview participant recalled, after receiving the SUAP funding, “*The floodgates opened, and we pretty much doubled our roster”* (SSP 1-202, Program admin). While the programs expanded enrollment, they soon realized they could not accept as many participants as originally planned. An allied health care provider at another program described the limits on enrolment as frustrating because:

“*We aimed for eighty-five clients per clinic in the first year. But it became quite clear […] we’d aimed too high. […] I think [this] was born out of eagerness to try and take on as many people as we could and as quickly as we could, but the reality is being staffed with three folks, one prescriber, one Registered Nurse, and one community health worker, we are [an] extremely small team”* (SSP 4-203, Allied health).

To manage the demand, programs modified their referral and intake procedures and created waitlists. This was not without challenges. One prescriber described how difficult it was to develop effective enrollment procedures as “*that’s the challenge of being a limited program in a huge sea of need*” (SSP 3-101, Prescriber NP). Some programs restricted intake calls to specific days and times of the week to respond to a flood of self-referrals and calls requesting admission for intimate partners and adult children. A nurse at one site said, their referral line* “was open five days a week, eight hours a day, [but] we would have way too many people to be able to go through, so we kept it very restricted, when you could call*” and subsequently “*people got upset”* (SSP 2-207, RN). This program later stopped taking phone referrals altogether and focused on in-person outreach:

*“Originally clients could call our phone line directly to be referred to or self-refer on the program. But now we’re deciding that, instead of doing it that way, we’re going to switch the focus more so to doing community outreach, to going to encampment sites and stuff like that to actually reach clients who are more—who [don’t have] access to phones, for example, or who have a harder time connecting”(*SSP 2-207, RN).

Another strategy the programs used to manage overwhelming demand was implementing a waitlist. Waitlists quickly created additional problems with more people being added to the waitlist than could be admitted into the programs. In some instances, the wait for admission exceeded two or three months, and service providers spoke about difficulties locating people when an enrolment spot opened. As a nurse lamented:

*“Our traditional population that we’re serving is people who struggle to access care through mainstream delivery models. […] we wanted to avoid having a really large waitlist because we’re noticing if you have a waitlist, folks don’t generally have a regular phone number, so the people who really need low-barrier entry are just lost at that point”* (SSP 4-101, Prescriber, NP).

The high overdose rates in the community led many service providers to worry about the consequences of wait times for admission. Providers expressed concern that waitlists created an expectation among potential clients that they would eventually be enrolled in the program. Furthermore, some providers worried a waitlist may have prevented potential program participants *“from seeking out other potentially life-saving addictions treatment, like methadone [or] suboxone*” if they expected they would be admitted to the SSP (SSP 2-101, Prescriber, Physician). This physician explained the waitlist could provide false hope as,* “right now there is just no movement on a waitlist, so it didn’t really make sense to give people that expectation.”* Service providers said there was little they could do to expand program capacity because of staffing and funding constraints, and as a result, waitlists were quickly abandoned in favour of same-day intakes. A prescriber described: “*We could easily add another hundred people on the wait list if we kept it up […] so we closed it […]. We need to figure something out where we can basically see someone right away*” (SSP 2–105, Prescriber, Physician).

### Creating a points-based admission criteria system

Existing practice guidance documents (Hales et al. 2019) did not include information to assist programs address admission pressure. As a result, programs had to develop localized admissions procedures and adjust them over time. This prescriber articulated the fluidity of the admission criteria, *“we just opened the gate and ran in, [we] tried to ramp up as fast as we could […] we are making stuff up as we go and fine-tuning stuff as we go” (SSP 3–101, Prescriber, NP*). For example, it was noted by service providers that many of the people first enrolled in the programs, particularly in Toronto, had well-established connections to supervised consumption sites and other harm reduction programs and therefore could more easily navigate admission into the SSP. This resulted in a lack of diversity among program participants. An allied health worker remarked:

“*At some point in the program when we saw that ninety percent of it is white and then a large portion of that is male. We wanted to be more inclusive of racialized folks, people from the LGBTQ2*+*, women, racialized from the LGBTQ2 community, people with disabilities*” (SSP 2-204, Allied Health).

In response, the programs developed a points-based system to guide decision-making. As a prescriber told us, the point system was developed,

“*To make sure that people who would traditionally have had difficulty accessing programs to get in […] We really wanted to tip the scale so that we could make sure we met the people who were having the biggest trouble with the overdose crisis*” (SSP 3-101, Prescriber, NP).

With equity concerns in mind, the prescribers described creating a system that assigned one point for different criteria and prioritized those with more points over those with fewer. These criteria included: frequent and recent history of opioid-related overdose; self-identifying as a woman, lesbian, gay, bisexual, two spirit, queer and two spirit (LGBTQ2S), Black, Indigenous, or racialized person; experiencing homelessness; and living with unmanaged HIV/HCV, palliative, sex worker, and/or pregnant. This admission points system was designed to prioritize those deemed most vulnerable for enrollment into the program. As one physician explained:

“*Pregnancy is always an immediate intake into the program, often same day if we’ve identified somebody, because we have such a shorter length of time to stabilize somebody’s use before we’re trying to get a healthy baby out, right?*” (SSP 1-101, Prescriber, Physician)

It was noted that these point systems effectively narrowed the admission criteria and increased admissions among certain high-risk groups. For example, an outreach worker concurred about priorities and indicated, *“We’ve done a lot of work recently with unhoused, pregnant women who find themselves sleeping rough. I would say probably in the last six to eight months we’ve probably rostered at least a dozen*” (SSP1 1–205, Allied health).

### Issues with the points-based admission criteria

Although the admission points system helped to refine priorities and decision-making, implementation challenges soon emerged. Service providers noted that the scoring system did not always reflect the complexity of potential participants’ needs and that *“it’s not a perfect system”* (SSP 2-102, Prescriber, NP). For example, a nurse explained,

*“It’s difficult when there are people who are housed [who] technically are only a one out of four [score], but arguably they need it more sometimes than people who haven’t overdosed in a long time, but still meet a three out of four [score] because they’ve had some kind of health complication in the past and they are homeless, so it’s difficult.”* (SSP 2-208, Registered Nurse)

Prescribers also worried that fully understanding the complexity of clients’ needs can take time and rapport, which could lead to screening out some people who did meet the more stringent criteria for program admission.

*“It’s just so reductive, right? You’re reducing people down to [..] boxes they check off […]. One [potential program participant] who would score initially at a 1, over time I’ve gotten to know that person and I’m, like, ‘Oh my god, you’re actually a 3, at least, but I turned you down […] we could have caught you earlier. While you were at a 1.’”* (SSP 3-201, , Prescriber, NP)

In contrast, a few service providers said some potential participants may have overstated their risk factors or medical conditions in the attempt to be triaged faster, to the frustration of service providers and potential participants. For example, a nurse described how although they understood the motivation behind such behaviour, overstating risk to match program admission criteria was something they tried to prevent. They explained it was *“something that I was conscious about at the beginning because I’ve also coached clients how to get into certain types of programs.”* They continued:

*“We’ve tried to prevent it from happening as best as possible. But of course, these things [program scoring criteria for admissions] get out. We’re not going —if someone scores in, it’s not as though we undo it. We wouldn’t go up to someone and say, ‘You fudged it here, you fudged it there, no.’ Anyone who uses opiates right now is at risk for overdose.”* (SSP 3-202, , Prescriber, NP)

This participant’s statement reflects the complexity of using a points-based admission system in the context of immense need and program demand.

### New circumstances create additional challenges

The emergent and evolving context of the COVID-19 pandemic created additional challenges for program capacity. In Toronto and London, COVID-19 isolation hotels were opened to provide unhoused people, who tested positive, a location to isolate and recover. Safer supply was prescribed to some people while in these hotels to prevent opioid withdrawal, premature departure and overdose while isolating. However, when discharged, these people either needed to find a prescriber in the community or return to using unregulated drugs. The safer supply prescribers in our study described feeling obligated to accept people who had started on safer supply elsewhere, even if they did not score high enough on the points system otherwise.

“*It’s been really challenging during COVID for capacity reasons as well – we’re still trying to apply these criteria but we’re also trying to take people on who are being started on safer supply in other places, particularly the COVID-positive recovery hotels, as well as the COVID-distancing hotels. We’re trying our best to take on a lot of folks, because they’re being titrated off their safer supply component of their medications when they leave […] It’s just a mess.”* (SSP 2-101, Prescriber, Physician)

Similarly, service providers told us they would also forgo the points system to enroll intimate partners of enrolled clients, particularly in the context of a participant sharing their medication or feeling pressured to do so by intimate partners. In these situations, prescribers believed it was best to also admit intimate partners who met basic criteria—history and imminent risk of overdose, history of enrolment in OAT—but would not necessarily score high on the current points system. As a nurse described, “*It’s really hard to treat one person in a couple if they’re sharing their medication with the other person*” (SSP 1-103, RN). While prioritizing the enrollment of intimate partners meant program participants no longer had to share their medications, it reduced enrollment spots in the program for others who might score higher on the admission criteria.

### Modifying the points system, high acuity and ever-increasing demand

Some programs continued to adapt and modify the points-based system in an iterative program implementation process. Some programs added weight to certain admission priorities to address concerns that a simple points system could obscure complexity. For example, a prescriber described that an infection complication from substance use (i.e., spinal abscess, endocarditis) was “*a ‘1’ and you get a plus if it’s something that’s a more serious infectious complication*” (SSP 2-105, Prescriber, Physician).

The revised point system effectively narrowed down who was admitted into the programs; however, as a result, only the highest acuity clients were admitted. *“Selecting for the most complex”* (SSP 3-202, Prescriber, NP) clients led to concerns and frustration among service providers. As this Nurse Practitioner explained, “*We are prioritizing folks who we are not optimized to serve […] the program itself wasn’t set up with those pieces in place*” (SSP 3-202, Prescriber, NP).

A significant challenge emerged from this new system. Programs described being unable to admit new program participants until they could stabilize recently admitted participants, which slowed admissions as participant complexity increased. Participants with complex health and social concerns typically required more frequent follow-up visits than those with less complexity, placing additional strain on program capacity. COVID-19 further increased complexity among program participants, and resources were simultaneously stretched during the height of the pandemic response.

*“I think we’ve probably hit the ceiling of how many people we can bring in until we start to stabilize the new intakes […] We’re in a very high acuity phase where, because of COVID and the suffering related to that, we brought in a lot of really complex patients […] People who are very marginalized—so people who continue to sleep rough or people with health conditions, like their HIV that we really need to get a good handle on, those people almost never come off of the weekly check-ins”* (SSP 1-101, Prescriber, Physician).

### Frustration, distress and burnout among service providers because of capacity issues

While providers often shared that their SSP work was extremely impactful and rewarding, most expressed distress resulting from the limited capacity of the programs and the policies they implemented to limit admissions. For example, a nurse practitioner said the point system “*works okay with a screening tool,”* but it was far from ideal as:

*“It’s causing a lot of personal and moral harm, I think. It’s just a trap to have to turn people away who desperately need the program. […] It’s not something we’re used to doing. And it’s hard to do” (*SSP 3-101, , Prescriber, NP).

Service providers described having to tell someone they did not meet the eligibility criteria as difficult: “*It was challenging when people didn’t meet criteria. Because a lot of people put a lot of hope on that phone call, and when they heard that they didn’t meet criteria, then it didn’t always go over very well.”* (SSP 2–207, RN). Not being able to enrol more participants had an emotional impact on service providers: “*It’s really tough. And they come in every week and—so sad, and still have all these problems and they’re still using fentanyl’*” (SSP 1–203, RN). Many service providers were morally opposed to restricting access to the programs but found themselves with few other options due to limited program capacity.

*“The reality is—being staffed with three folks per clinic […] we have no backup for sick days and vacation. It’s been quite a journey and a learning process to figure out how to really be realistic about our capacity and also to start integrating more supports for staff, so that we can avoid burnout.”* (SSP 4-103, Allied Health)

Many service providers felt responsible for the consequences of not admitting someone to the program, such as a person experiencing an overdose or dying which further added to service providers’ moral distress. For example, as this physician explained:

*“I definitely feel there’s a lot of fatigue and exhaustion and burnout. […] A number of people on our waitlist, actually while waiting for the program, ended up dying from overdose. So, it’s just such a messy space to be in, and it’s exhausting.”* (SSP 2-105, Prescriber, Physician)

Due to the limited capacity of the programs, service providers described feeling exhausted from working long hours: “*We just end up working till nine p.m., and exhausted and drained,”* which could further reduce the capacity to admit new participants: “*this then ends up having a broader impact on intake capacity and feeling like we may not have capacity to take on folks*” (SSP 2-105, Prescriber, Physician). As one way of addressing the small number of prescribers in Toronto, an ‘on-call’ system was implemented to ensure continuity of opioid dispensing and avoid disruptions for program participants. Prescribers took turns responding to off-hour calls from pharmacists to answer questions or address dispensing/prescribing problems. While this was done so that prescribers only had to be ‘on-call’ one weekend each month, it further added to their exhaustion.

Many service providers were disappointed that they were among the few prescribers willing to use this new approach to address the complex problem of fentanyl-related overdose. Some felt ostracized, professionally attacked by or isolated from their colleagues. Some participants described feeling unsupported by colleagues who prescribed opioid agonist treatment, as they had relevant clinical experience and the ability to prescribe safer supply but most refused to do so. One prescriber likened their experience to a time when few physicians were willing to provide care to people living with HIV:

*“We take over clients of other well-known methadone providers in the city who are really against the program, and we see how terrible the care has been and just how easy [sic] it could actually be shifted. […] I relate it similarly to how I think clinicians must have felt when the HIV crisis first happened, where people were listening to community members who were demanding support and care and only a handful of people within the system actually stepped up in the beginning. And it just took years and years of so much advocacy to actually normalize HIV care.” (*SSP 3-105, Prescriber, Physician)

### Managing the impact of capacity issues on service providers

Service providers agreed that no admission policy (e.g., waitlists, points-based systems) could be designed to meet the ever-increasing demand in the community. An advocacy toolkit was developed to help manage service providers’ distress and address the overwhelming demand for people who had sought admission to a SSP but had not been admitted. The toolkit provided information to support people in advocating for prescribed safer supply from a physician who prescribed OAT and was intended to ensure that those unable to get into a SSP were still provided with some resources. The toolkit was described as including:

*“.. a handout around guidelines coming from [British Columbia] as well as a guideline we created around how to prescribe. And [we] encourage people to take that to their current OAT clinician, and if they didn’t have a current OAT[prescriber] we also gave a handout around more established and better clinics in the city that see people who use drugs.”* (SSP 3-0101, Prescriber, Physician)

However, most service providers said that to truly meet the perceived demand, SSPs needed to expand beyond “*a boutique program”* (SSP 1-101, prescriber, physician). Participants suggested that demand could be better met by structural change such as increasing prescribing capacity (e.g., increased number of prescribers, expansion into primary care), greater support from addiction medicine clinicians, different models of service delivery including non-medicalized and community-led compassion clubs and improving the geographical distribution of the programs. However, as this nurse indicated, these structural changes would require significant buy-in from multiple groups:

*“Getting enough prescribers to be able to put a dent into the problem is really necessary. So, more advocacy within providers and more buy-in, which, in order to get that we need the regulatory bodies to buy into it and government to buy into it, and the community to buy into it. […] I think there’s a lot of fear among providers and these programs aren’t big enough to deal with the problem. And so really, really expanding the programs is very necessary, but there’s a lot of convincing to do before that happens.”* (SSP 2-207, RN)

Additionally, some participants acknowledged that safer supply programs could only ever be one facet of addressing the crisis and there is a need for a system-wide approach to addressing overdose deaths. As this physician said, they would like to see “*higher-upstream level of work that’s truly transformative, and gets at the root of these issues, as opposed to what feels like a really downstream intervention, just trying to keep people alive*” (SSP 2-105, Prescriber, Physician). For some, this meant that they did not feel they could ever fully address the issue clinically, and instead regulation, decriminalization or legalization could better address the consequences of a volatile unregulated drug supply.

*“We’re dealing with all these clinical consequences of the changing supply. Maybe if we had the capacity to put everybody on, then we could get ahead of the street supply but I don’t think that’s possible. There’s not enough doctors in Canada who will ever be willing to do this. And so my messaging, I think, has changed over five years from ‘safe supply for everybody’ to ‘safe supply can’t be done by doctors, we need government to legalize and regulate.’”* (SSP 1-101, Prescriber, Physician)

## Discussion

**Fig. 1:**
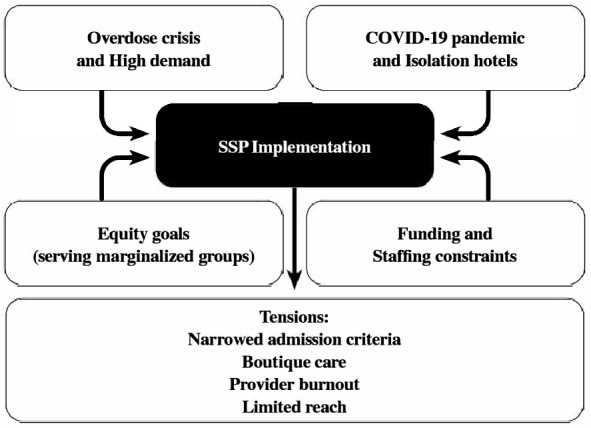
Tensions and constraints shaping safer supply program (SSP) implementation

Our findings demonstrate that the SSPs are complex interventions that needed to adjust and adapt during their implementation within complex circumstances. SSPs were implemented as an emergency intervention, and their implementation was shaped within the context of two rapidly shifting public health emergencies (i.e., overdoses from the unregulated drug supply and COVID-19). As a result, service providers found themselves having to refine the program as it was being implemented (see Fig. 1). The urgent demand for overdose prevention services conflicted with the programs’ limited capacity, leading to a gradual tightening of admission criteria to manage access. This tightening resulted in admitting increasingly medically complex clients, who required more intensive resources (e.g., shorter, more frequent appointments) over extended periods to stabilize. As a result, the programs had a diminished capacity to enroll new participants. This in turn created challenges for the reach and sustainability of the programs, contributed to burnout amongst service providers, and limited the overall impact of SSPs at the population level.

There has historically been a lack of diversity among people who access harm-reduction interventions. Men are over-represented in harm reduction program utilization with women historically being underserved [51, 52]. Most harm reduction programs have been designed around the experiences of white men and often lack women-specific supports like childcare, trauma-informed care, or women-only spaces. Intersectional barriers—including racism, poverty, and social control by partners—further limit women’s access and engagement. Stigma, fear of child apprehension, and gender-based violence, which make services feel unsafe or unwelcoming, add to the under-representation of women in these programs. However, within the London, Ontario SSP, which had been operating the longest, the majority of clients both before and after the pilot funding were women [18, 53]; however, when the Toronto programs were first implemented, they struggled to ensure equity among participants. Because the pilot funding led to an immediate influx of clients in Toronto, initially enrollment favoured those already well represented in harm reduction services, such as white men for the reasons noted above. To address equity issues, the programs revised their points criteria to prioritize those most often excluded from harm reduction interventions. However, this shift further narrowed the SSPs scope, transforming them into highly targeted interventions for marginalized populations without the requisite funding to meet their complex needs. While this prioritization addressed critical service gaps, it likely came at the cost of reaching fewer people. Pauly, McCall [54] used concept mapping with people who use drugs to inform safer supply program planning and reported that participants emphasized that ease of access was part of effective programs. By attempting to address equity issues, the programs potentially, and paradoxically reduced the potential impact on overdose mortality at a population level.

Within the framework of ‘evidence-based practice,’ interventions are often depicted as stable entities, evidenced using a hierarchy of controlled conditions such as randomized controlled trials. Implementation science is concerned with how such interventions are implemented within specific external contexts [40, 55]. Rhodes et al. suggest instead that context not only shapes an intervention, but interventions shape context through dynamic, situated knowledge-making practices [40]. Their ‘evidence-making’ framework, in contrast to ‘evidence-based,’ draws attention to “the process and practices through which ‘evidence’ ‘intervention’ and ‘context’ come to be” [40]. SSPs were not merely adapted in a local context, they were transformed while they were being enacted. Through dynamic intra-action, both the safer supply intervention *and* the evidence for how to operate the programs were co-constituted through the local processes of intervening. For example, to create and refine admission criteria, prescribers drew on multiple sources of knowledge including professional experience, practice guidelines, and new evidence that emerged through the process of making and remaking program policies. In the evolving context of funding and staffing constraints, COVID-19, and escalating overdose rates, staff struggled to establish and implement admission procedures that adequately met the needs of their programs. Through this process, what was envisioned as a system-level response evolved into a boutique model of care, tailored to the most marginalized individuals but not adequately resourced to meet the needs of the program participants.

The participants in our study uniformly noted that the funding from Health Canada was welcome but inadequate. Due to limited resources, programs were unable to hire additional staff to meet growing demand or address inequities in access. The added strain of the pandemic further stretched existing resources, leaving service providers feeling overwhelmed and exhausted. Similarly, in their thematic analysis of 45 progress reports from 11 pilot programs across Canada that received Health Canada funding, Karamouzian, Rafat [24] noted that a high staff-to-client ratio coupled with limited and short-term funding and the external pressures of the pandemic resulted in significant staffing issues, high staff turnover, and burnout among service providers. Healthcare workforce challenges, including shortages of general practitioners and nurses complicate the feasibility of scaling SSPs, and our results highlight the challenges of implementing innovative public health interventions in an under-resourced primary care system. Medicalized models of safer supply are resource-intensive and costly, making them difficult to scale without significant adaptations [24]. In Ontario, between 2016 and 2020, 155 clinicians prescribed safer supply, over 80% of whom were general practitioners [22]; however the number of prescribers in Ontario remains small, and it is unclear if individual physicians and small pilot programs are sufficient to meet the ever-growing demand in the face of an increasingly toxic unregulated drug supply.

Effectively addressing the toxic and volatile unregulated drug supply will require us to reimagine safer supply beyond boutique care. However, opposition to safer supply in Canada has grown since conducting our interviews, fueled by media narratives and political rhetoric suggesting they are ineffective and contribute to opioid dependence [56]. Opposition is also linked to concerns about the diversion of safe supply medications into the illicit market, raising tensions between public health goals of harm reduction and concerns about unintended community-level risks. Evidence shows varied factors drives sharing or selling among peers of safe supply medications including inadequate dosing of hydromorphone relative to unregulated fentanyl, barriers to program access, and poverty [57, 58]. Inadequate dosing can be addressed by providing more drug options, including powder fentanyl, which is only available in one safer supply program in Vancouver, Canada [59]. At the time of this study, SSPs were funded by Health Canada through its SUAP program; funding ended in March 2025. In preparation for closure, most SSPs transferred as many patients as possible to individual primary care providers willing to continue prescribing safe supply medications but without funding for wrap-around supports or to transition willing patients to OAT service providers (personal communication). The impact of these transfers on SSP patients is unknown, but several studies on the subject are currently underway in Canada. A few SSPs continue to prescribe to patients albeit without funding for case managers or other supports (personal communication). In March 2023, Alberta implemented a policy that effectively banned most prescribed safer supply programs by restricting such prescribing to a few high-barrier “Narcotic Transition Services” clinics with strict protocols [60]. This decision, driven by regulatory changes and political opposition, cut off access for many and has been widely criticized for limiting harm reduction options in the province. In Ontario, the current government has openly opposed harm reduction initiatives and shifted its focus away from safe consumption sites to an addiction recovery hub model that will explicitly “not offer ‘safer’ supply” [61, 62]. As a result, many supervised consumption sites have closed in Ontario. The feasibility of non-medical models of SSPs in Canada is also questionable as policymakers have shown little willingness to formally support non-prescriber-based models, such as compassion clubs that provide alternatives to the volatile unregulated drug supply. For example, a Vancouver-based compassion club operating since 2020 was raided [63], and the provincial government in British Columbia declined to heed the Chief Coroner’s call for expanded non-prescriber alternatives to the toxic unregulated drug supply [64].

The SSPs in our study were small pilot programs that evolved through implementation within a complex environment with significant unknown factors. Through implementation in medicalized SSPs, ‘safer supply’ has come to represent not only practices of prescribing alternatives to the unregulated drug supply, but also resource-intensive wrap-around care for marginalized people who use drugs. The ‘story of evidence’ for SSPs is of an emergent evidence-making intervention made and remade with a social, cultural and political context that restrained the possibilities of what SSPs could become before they could be evidenced in ways that meet standards of ‘rigour’ within the framework of ‘evidence-based’ interventions. The accessibility challenges of primary care-based SSPs reported in our findings should not be taken as evidence that such programs or the entire concept of safer supply are not feasible. By shifting the focus of implementation to process, rather than outcomes, an evidence-making framework invites speculation as to how interventions could be done differently [65]. Viewing SSPs in this way encourages us to consider questions about the effects of intervening, or perhaps more importantly not intervening within a public health emergency.

Our study has several limitations. We describe the evolution of admission criteria shortly after the programs received pilot funding and as a result, we provide a brief snapshot of the implementation of SSPs. Our results do not describe the many adaptations that have been made since our interviews were conducted. Our interviews were also conducted during the height of the COVID-19 pandemic when there was greater support for emergency interventions [66]. SSPs likely experience different issues regarding admission policies as opposition to these programs has intensified. Our study included only four SSPS, three of which were in Toronto, and there could be different implementation experiences based on a multitude of factors including program location and duration of operation before pilot funding. Nonetheless, our study offers valuable insights into the adaptations implemented to address high demand for a program in a resource-limited setting.

## Conclusion

The implementation of SSPs in Ontario and the closure of three of the four programs we studied highlights the challenges of addressing intersecting public health emergencies in a resource-constrained healthcare system and amidst political opposition. SSPs were adaptive and evolved in real time; while these adaptations addressed significant equity gaps, they also underscored the limitations of operating within an under-funded primary care model. The narrowing of admission criteria, necessitated by overwhelming demand and limited resources, shifted SSPs from broader harm reduction interventions to boutique care, ultimately constraining their reach and population-level impact.

## Data Availability

The datasets generated and/or analysed during the current study are not publicly available at the request of the participating sites and participants given the sensitive nature of the data but may be available from the corresponding author on reasonable request.

## Abbreviations

CFIR: Consolidated framework for implementation research
COVID-19: Coronavirus disease 2019
HCV: Hepatitis C
HIV: Human immunodeficiency virus
LGBTQ2S: Lesbian, gay, bisexual, two spirit, queer and two spirit
MAXQDA: MAXimum qualitative data analysis
NP: Nurse practitioner
OAT: Opioid agonist therapy
RN: Registered nurse
SPSS: Statistical package for the social sciences
SSP: Safer supply programs
SUAP: Substance use and addiction program, health Canada
