# Correction: A sea of need: provider accounts of strategies used to manage admission demands to safer opioid supply programs in Ontario

**DOI:** 10.1186/s12954-026-01473-3

**Published:** 2026-06-01

**Authors:** Carol Strike, Katherine Rudzinski, Rose A. Schmidt, Gillian Kolla, David Kryszajtys, Melissa Perri, Nat Kaminski, Adrian Guta

**Affiliations:** 1 Dalla Lana School of Public Health, University of Toronto, 155 College StRoom 500, Toronto, ON M5T 3M7 Canada; 2 Faculty of Medicine, Memorial University, St. John’s, Canada; 3 School of Social Work, University of Windsor, Windsor, Canada

**Correction to: Harm Reduct J (2025) 22:194** 10.1186/s12954-025-01344-3

In this article [1], the author’s name David Kryszajtys was incorrectly written as David Kryszajits. The original article has been corrected.
